# Effects of the signaling molecule cyclic-di-GMP on cyanobacterial circadian rhythm in *Synechococcus elongatus* PCC 7942

**DOI:** 10.1128/jb.00574-25

**Published:** 2026-02-27

**Authors:** Chihiro C. Yamaguchi, Robert A. Kanaly, Eri Nishizaki, Keiichi Yamashita, Koichiro Takatsuki, Yoshihiko Furuike, Mingxu Fang, Shuji Akiyama, Setsuyuki Aoki, Masaki Tsukamoto, Yoichi Nakahira, Susan S. Golden, Shinsuke Kutsuna

**Affiliations:** 1 Department of Life and Environmental System Science, Graduate School of Nanobioscience, Yokohama City University 199553, Yokohama, Kanagawa, Japan; 2 Division of Trans-Hierarchical Molecular Systems, Research Center of Integrative Molecular Systems (CIMoS), Institute for Molecular Science, National Institutes of Natural Sciences 88301, Okazaki, Japan; 3 Center for Circadian Biology, University of California, San Diego 8784, La Jolla, California, USA; 4 Graduate School of Informatics, Nagoya University, Nagoya, Aichi, Japan; 5 College of Agriculture, Ibaraki University 98282 , Ami, Ibaraki, Japan; 6 Faculty of Applied Biological Science, Ibaraki University 12819, Ami, Ibaraki, Japan; 7 United Graduate School of Agricultural Science, Tokyo University of Agriculture and Technology, Fuchu, Tokyo, Japan; 8 Department of Molecular Biology, University of California, San Diego 8784, La Jolla, California, USA; Queen Mary University of London, London, United Kingdom

**Keywords:** *Synechococcus elongatus*, c-di-GMP, cyanobacteria, circadian clock, *ydeH*, *yhjH*

## Abstract

In phototrophic cyanobacteria, the signaling molecule bis-(3′-5′)-cyclic dimeric-guanosine monophosphate (c-di-GMP) plays important roles in a wide variety of functions associated with environmental conditions, including biofilm formation, motility, heterocyst development, cell size control, phototaxis, and flocculation. However, its role in circadian rhythms, which contribute to fitness under diel light conditions, remains unexplored. In this study, we investigated the impact of changes in intracellular c-di-GMP levels on the circadian clock of the model cyanobacterium *Synechococcus elongatus* PCC 7942. Using inducible expression systems for the *Escherichia coli* genes *yhjH* (encoding a c-di-GMP phosphodiesterase) and *ydeH* (encoding a diguanylate cyclase), we modulated intracellular c-di-GMP concentrations. The effects were analyzed by monitoring bioluminescence rhythms from a luciferase reporter under the control of the clock. Induction of *yhjH* lengthened the circadian period by 0.6 h, while *ydeH* induction caused a phase delay of 5.8 h and reduced peak amplitude on the third circadian day. Additionally, exogenous c-di-GMP administration at the onset of darkness in a 12-h light/12-h dark cycle delayed the peak of the rhythm by 1 h, whereas administration at light onset had no effect. These results demonstrate that c-di-GMP levels in *S. elongatus* influenced multiple properties of the cyanobacterial circadian clock, including period, phase, and amplitude during light–dark cycles, suggesting that c-di-GMP signaling networks likely integrate environmental cues into circadian regulation through as yet unidentified factors.

**IMPORTANCE:**

bis-(3′-5′)-Cyclic dimeric-guanosine monophosphate (c-di-GMP) is a widely conserved bacterial signaling molecule that controls diverse physiological processes such as biofilm formation and motility, yet its influence on the circadian clock in cyanobacteria has not been described. Using inducible expression of *E. coli* enzymes and exogenous administration to alter intracellular c-di-GMP in a model cyanobacterium *Synechococcus elongatus* PCC 7942, we demonstrate that c-di-GMP modulates key properties of the circadian clock, including period, phase, and amplitude. These findings uncover a novel regulatory layer linking bacterial signaling networks to circadian clock regulation in cyanobacteria and suggest that c-di-GMP signaling enhances cyanobacterial fitness in natural light–dark cycles by fine-tuning circadian dynamics.

## INTRODUCTION

In response to extracellular stimuli, intracellular signaling molecules are produced in bacterial cells. Nucleotide derivatives such as guanosine pentaphosphate, bis-(3′-5′)-cyclic dimeric-adenosine monophosphate (c-di-AMP), and bis-(3′-5′)-cyclic dimeric-guanosine monophosphate (c-di-GMP) are important signaling molecules in phototaxis, photosystem repair, dark survival, and other behavioral characteristics in cyanobacteria (1–3).

c-di-GMP was first identified as a factor promoting cellulose biosynthesis in *Gluconacetobacter xylinus* (formerly *Acetobacter xylinum*) and is recognized as a ubiquitous bacterial signaling molecule that regulates cell motility, virulence, cell differentiation, and peptidoglycan synthesis (4–8). c-di-GMP is synthesized from two molecules of GTP by diguanylate cyclases (DGCs) that harbor the conserved GGDEF catalytic motif (9), whereas its degradation to pGpG or GMP is mediated by phosphodiesterases (PDEs) containing EAL or HD-GYP domains (10, 11).

Most cyanobacterial genomes examined to date are inferred to contain genes encoding proteins with the GGDEF motif and/or the EAL domain, as well as the HD-GYP domain, indicating that c-di-GMP plays important roles in cyanobacteria (12). Indeed, c-di-GMP levels influence cell motility and biofilm formation, as was demonstrated in a study of the GGDEF protein-encoding gene *dgc2* in the filamentous cyanobacterium *Leptolyngbya boryana* (8). Another filamentous species, *Anabaena* sp. PCC 7120 develops nitrogen-fixing heterocysts, in which the regulation of c-di-GMP levels is crucial for controlling heterocyst development and cell size through the activity of DGCs with the GGDEF motif (CdgS [13]). Moreover, CdgSH, a bifunctional enzyme that has the GGDEF motif and EAL domain, synthesizes and degrades c-di-GMP, which may be required to control heterocyst development, in addition to the activity of CdgS (14). To control cell size, CdgS also functions as a response regulator in a two-component signaling system, synthesizing c-di-GMP in response to the histidine kinase CdgK. The resulting c-di-GMP levels are sensed directly by the c-di-GMP receptor, CdgR, and the signaling pathway involving CdgK, CdgS, and CdgR interacts directly with the global transcription factor DevH to regulate cell size (15, 16).

 In *Synechocystis* sp. PCC 6803, the DGC Cph2, which contains multiple light-sensing GAF domains, synthesizes c-di-GMP under blue light, and this Cph2-dependent elevation of c-di-GMP inhibits motility (17). The thermophilic cyanobacterium *Thermosynechococcus vulcanus* exhibits light-quality-dependent aggregation and motility, which are regulated by three photoreceptors—SesA (harboring a GGDEF motif), SesB (harboring an EAL domain), and SesC (harboring both)—that together coordinate cellular c-di-GMP levels (18). In *Synechococcus elongatus* PCC 7942, hereafter *S. elongatus*, 20 genes encode proteins associated with GGDEF, EAL, and HD-GYP domains. The GGDEF/EAL protein SL2 contains a light-receptor LOV domain and degrades c-di-GMP under blue light conditions *in vitro* (12, 19). However, the physiological functions of these genes in *S. elongatus* have remained unclear.

Cyanobacteria such as *Synechococcus* spp. possess circadian clocks that regulate nitrogen fixation, amino acid uptake, cell division, and gene expression (20). In *S. elongatus*, the circadian clock is composed of KaiA, KaiB, and KaiC (21). KaiC undergoes cycles of autophosphorylation and autodephosphorylation, and these reactions are regulated by KaiA and KaiB, respectively (20, 22). Interestingly, the phosphorylation state of KaiC affects the availability of binding sites for KaiA and KaiB, thereby controlling the progression cycle over a circadian period (23).

To enhance fitness under diel light–dark cycles, the cyanobacterial circadian clock is modulated through control of clock-associated genes and proteins, such that the timings of the outputs, e.g., cell division and nitrogen fixation, are determined by peak and trough timings (i.e., circadian phases) (20). Indeed, in response to the environmental transition from light to dark, cyanobacterial circadian clocks reset the state of their core oscillator, that is, the KaiC phosphorylation cycle as mentioned above (24). The resetting is mediated in part by changes in the cellular ADP/ATP ratio that arise from the light dependence of photosynthetic ATP production (25). The phosphorylation status of KaiC is transmitted to the DNA-binding response regulator RpaA via the cognate histidine kinase SasA, ultimately generating a genome-wide circadian transcriptional rhythm (26).

SasA and CikA further fine-tune this ADP/ATP-induced resetting of the KaiC phosphorylation cycle (24). Environmental light–dark transitions also alter the oxidation state of plastoquinone, which binds to KaiA and modulates the KaiC phosphorylation cycle (27–29). Light–dark transitions additionally induce the accumulation of Pex, a repressor of the *kaiA* gene; in the absence of Pex, the circadian phase may advance (30). This finding highlights the importance of transcriptional regulation of *kaiA* by environmental light signals in determining circadian phasing (31, 32).

In this study, we investigated whether c-di-GMP influences circadian clock function in the cyanobacterium *S. elongatus* PCC 7942 by continuously or transiently inducing *Escherichia coli* PDE- and DGC-encoding genes, *yhjH* and *ydeH*, respectively, as well as by administering c-di-GMP directly to cell cultures. Results suggest that intracellular maintenance of c-di-GMP levels is important to the circadian clock period, phase, and amplitude during light–dark cycles.

## RESULTS

### Induced heterologous expression of *yhjH* extends the circadian period

**Fig 1:**
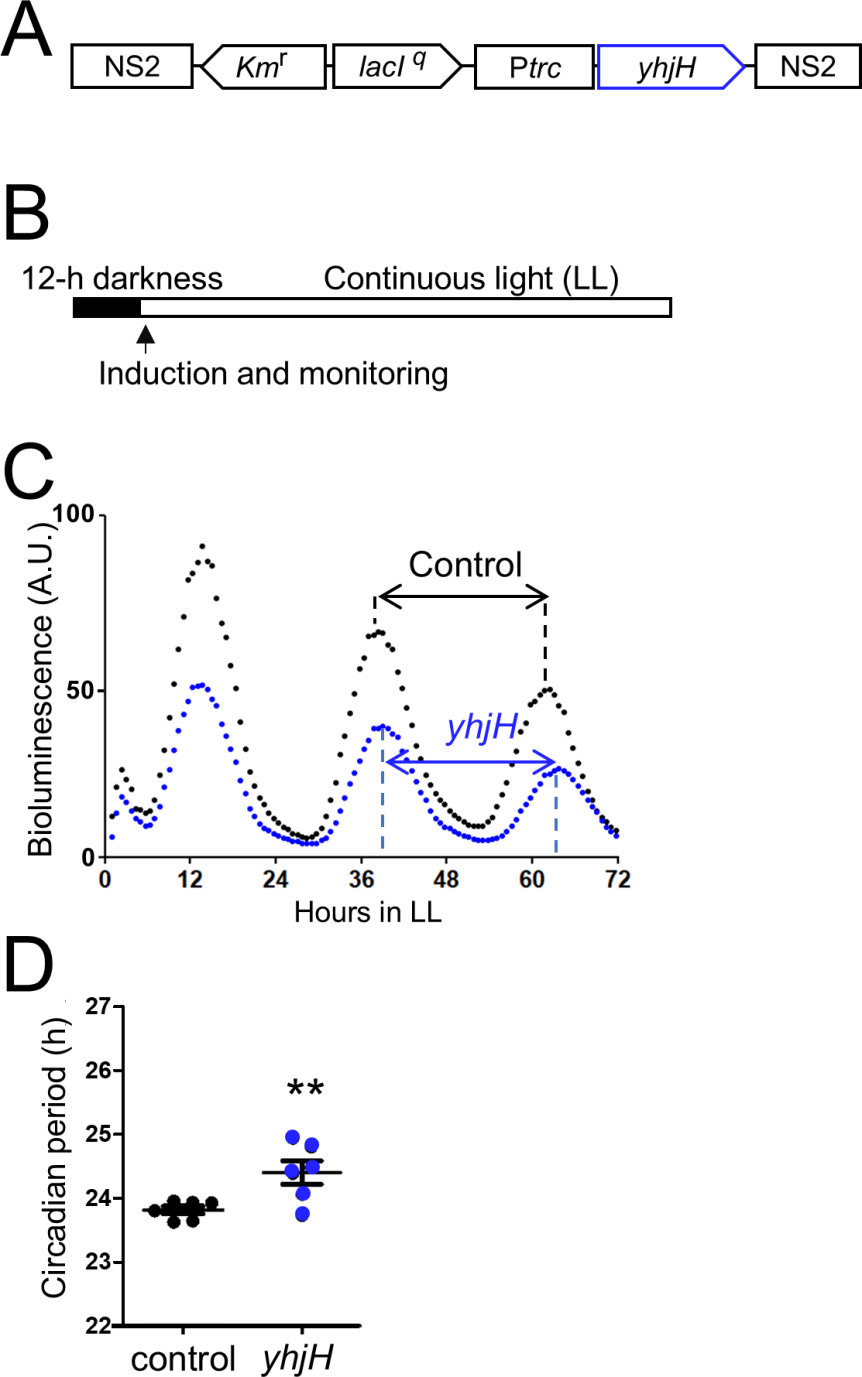
Circadian period extension caused by induction of *yhjH*, an *E. coli* gene encoding a c-di-GMP degradation enzyme. (**A**) Schematic of the inducible *yhjH* gene fusion P*trc::yhjH* in the *S. elongatus* genome region NS2. *lacI^q^*, a gene encoding the repressor for the *trc* promoter (P*trc*). *yhjH* is under the control of the *E. coli trc* promoter, repressed by LaqI^q^ and derepressed by the addition of isopropyl-β-D-thiogalactopyranoside (IPTG). (**B**) Cultures grown under continuous light were subjected to 12-h darkness (filled bar). The arrow indicates the administration of the inducer IPTG (50 µM) at the onset of the light. Bioluminescence monitoring was performed under light conditions (white bar, LL). (**C**) Representative bioluminescence rhythms of reporter strains expressing *yhjH* under control of the IPTG-inducible *trc* promoter. IPTG was administered at 50 μM. A.U., arbitrary unit; control, strains harboring *trc* promoter without *yhjH*; *yhjH*, *yhjH-*expressing strains. Two-way arrows indicate circadian periods. (**D**) Distributions of the circadian periods. Error bars represent SEM. **, *P* < 0.01 (Student’s *t*-test, *n* = 6).

To test whether a decrease in intracellular c-di-GMP affected the circadian clock in *S. elongatus*, we utilized a bioluminescent *S. elongatus* strain, NUC42 (32), transformed with the *E. coli yhjH* gene, which encodes the c-di-GMP-specific phosphodiesterase YhjH (18) (Fig. 1A). Under induction of gene expression (Fig. 1B), we measured the circadian rhythms (parameters defined in Fig. S1) of a *yhjH*-expressing strain and a control strain that did not harbor the *yhjH* gene (Fig. 1C). The bioluminescence rhythm in the control strains exhibited a circadian period of 23.8 ± 0.1 h, and the *yhjH*-expressing strain exhibited a circadian period of 24.5 ± 0.4 h under induction conditions (Fig. 1D). These results showed that the period was prolonged by 0.6 h after induction of *yhjH* gene expression. When another modified strain with a null mutation in the YhjH-expressing gene was investigated (amino acid residues for the catalytic motif of YhjH were altered from Glu-Leu-Leu to Ala-Leu-Ala), it was found that the period length was identical to the control (Table 1). These results suggested that the enzymatic activity of YhjH c-di-GMP phosphodiesterase influenced the circadian period.

**TABLE 1:** Circadian period lengths of different *yhjH*-expressing strains^*a*^

Strain	Period (h)	SD	*P* value
P*_trc_*	24.7	0.4	–^*b*^
P*_trc_*::*yhjH*	25.5	0.3	0.03*
P*_trc_*::*yhjH*^ALA^	24.7	0.2	0.45

^*a*^ Asterisk shows statistical significance. Student’s *t*-test (*n* = 3).

^*b*^ –, indicates that no values were available.

### Temporal induction of *yhjH* expression delays circadian peak phase timing

**Fig 2:**
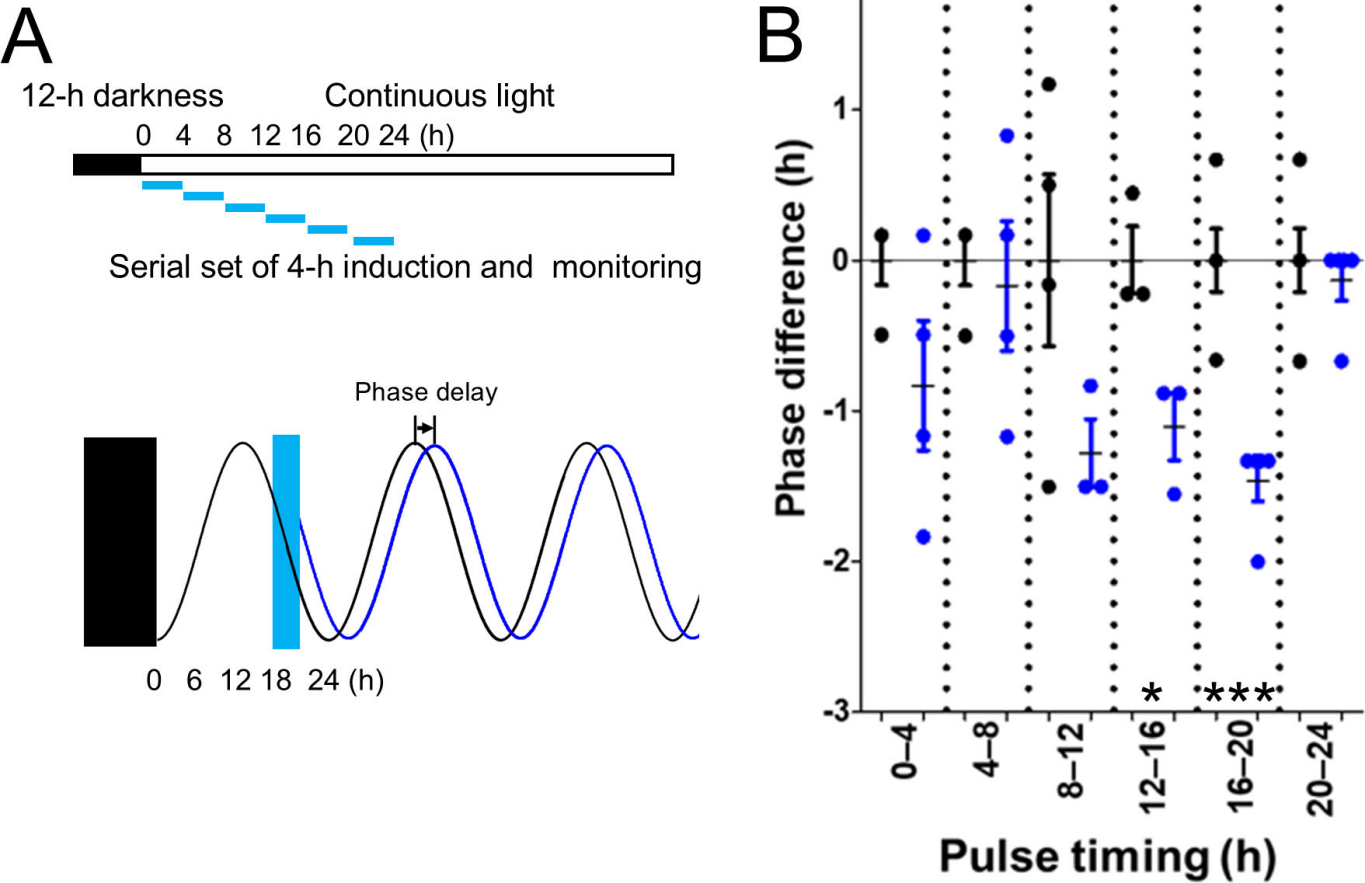
Circadian-phase delay induced by pulse expression of *yhjH*. (**A**) Experimental procedure and induced phase delay of the rhythm. Cultures grown under light were subjected to 12-h darkness (filled bar and rectangle). Pale blue boxes indicate 4-h induction periods of *yhjH*, followed by IPTG removal via medium exchange. Bioluminescence monitoring was conducted under continuous light. The schematized phase-delay rhythm, which resulted from induction between 16 and 20 h, is shown in black (control strain) and blue lines (*yhjH* strain). (**B**) Distribution of circadian-phase differences based on peak timing between *yhjH* and control strains. The vertical axis represents the distribution of the circadian phase. Control averages (circles with black lines) were set to 0 h; peak timings in *yhjH* strains are shown as cyan circles. Positive values indicate phase advances; negative values indicate delays. Error bars represent SEM. *, *P* < 0.05; ***, *P* < 0.001 (Student’s *t*-test, *n* = 3).

To probe which phase of the circadian clock was changed during circadian period extension, *yhjH* expression was induced by 4-h pulse treatments for 0–24 h under light conditions, as shown in Fig. 2A, and circadian rhythms were measured. No effect was detected by the 4-h pulse inductions for 0–12 h and 20–24 h; however, for 12–20 h, the inductions caused a phase delay of 1.1 ± 0.2 h (Fig. 2B). These results supported the hypothesis that the circadian clock of *S. elongatus* was sensitive to c-di-GMP reduction through degradation at certain timings, i.e., from the beginning to the middle of the subjective night of *S. elongatus*.

### *yhjH* gene induction caused changes to intracellular c-di-GMP levels in *S. elongatus*

Considering that the results of the above experiments showed period extension and phase delay, intracellular c-di-GMP levels were quantified in *S. elongatus* (Fig. S2). In response to dark-to-light transitions at 0 h, the control strain, which carried the same inducible promoter as in the constructs but without *yhjH*, resulted in a decrease in c-di-GMP levels by approximately 33% after 2 and 4 h (Fig. S2A). This certain decrease is a light response in *S. elongatus*, whose physiological role is unknown (33). In the *yhjH* stain, c-di-GMP levels further decreased to less than half during the induction of *yhjH* (Fig. S2A; P*trc::yhjH*). Thus, a greater decrease in c-di-GMP was observed in the *yhjH* strain compared to the control, indicating that induction of *yhjH* is responsible for the reduction in c-di-GMP levels. However, from 12 to 48 h after induction, c-di-GMP levels in the *yhjH* strain declined to a similar extent as those in the control (Fig. S2B), suggesting that the assay had reached its detection limit. Given that the observed effect on circadian period length was limited to only 0.6 h, the reduction in c-di-GMP in the *yhjH* strain appears to be minimal relative to the control.

### Induced heterologous expression of *ydeH* delays the circadian phase

**Fig 3:**
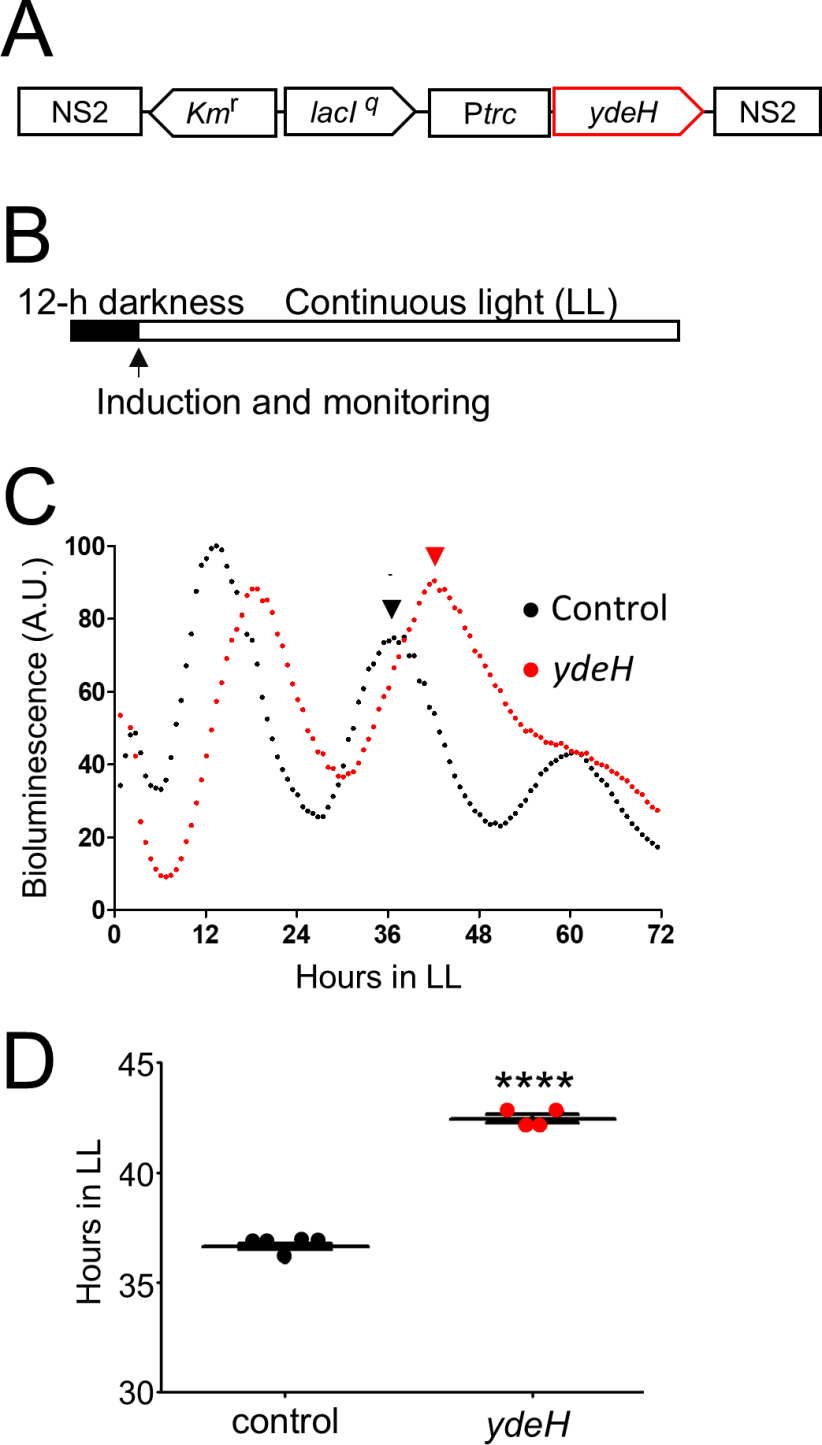
Circadian-phase delay caused by induction of *ydeH*, a gene encoding a c-di-GMP synthesis enzyme. (**A**) The inducible *ydeH* gene fusion P*trc::ydeH* in the *S. elongatus* genome region NS2. (**B**) Experimental procedure and light conditions. The culture grown in light condition was subjected to 12-h darkness (black bar). The arrow indicates the timing of administration of the inducer IPTG at the onset of the light period. Bioluminescence monitoring was carried out for the following 72-h light conditions (LL). (**C**) Representative bioluminescence rhythms of reporter strains harboring *ydeH*. IPTG was administered at 50 µM. Control, control strain harboring *trc* promoter without *ydeH*; *ydeH*, *ydeH*-expressing strain. Arrowheads mark peak timing. (**D**) Distributions of the peak timing. Error bars represent SEM. ****, *P* < 0.0001 (Student’s *t*-test, *n* = 4–5).

To assess the effect of elevated intracellular c-di-GMP on circadian rhythms in *S. elongatus*, the bioluminescence strain was transformed with *ydeH*, which encodes a c-di-GMP-specific DGC (18) (Fig. 3A); under induction, intracellular c-di-GMP increased fivefold by 4 h, when compared to levels before induction (Fig. S2A). The bioluminescence rhythm of the *ydeH*-expressing strain was measured under induction conditions. The peak phase was delayed by 5.8 ± 0.2 h (mean ± SEM, *n* = 4, *P* < 0.001, Student’s *t*-test), and subsequently, peak amplitude clearly decreased (Fig. 3A through D). To confirm that the DGC catalytic activity of YdeH accounts for the phase effects, we examined the rhythm of another strain, *ydeH*^GGAAF^. This strain expresses a modified YdeH protein in which the catalytic site residues (Gly-Gly-Glu-Glu-Phe) are substituted with Gly-Gly-Ala-Ala-Phe, resulting in loss of enzymatic activity. The rhythm in the *ydeH*^GGAAF^ strain exhibited the same phase response as the control strain (Table 2). These results suggest that the diguanylate cyclase activity of YdeH is responsible for the observed circadian-phase delay.

**TABLE 2:** Circadian phase of different *ydeH*-expressing strains^*a*^

Strain	Phase (second peak) (h)	SD	*P* value
P*_trc_*	34.7	0.7	–^*b*^
P*_trc_*::*ydeH*	38.4	0.8	6.2 (10^−5^)*
P*_trc_*::*ydeH*^GGAAF^	34.8	0.3	0.36

^*a*^ Asterisk shows statistical significance. Student’s *t*-test (*n* = 5).

^*b*^ –, indicates that no values were available.

### Temporal induction of *ydeH* gene expression advances circadian peak timings

**Fig 4:**
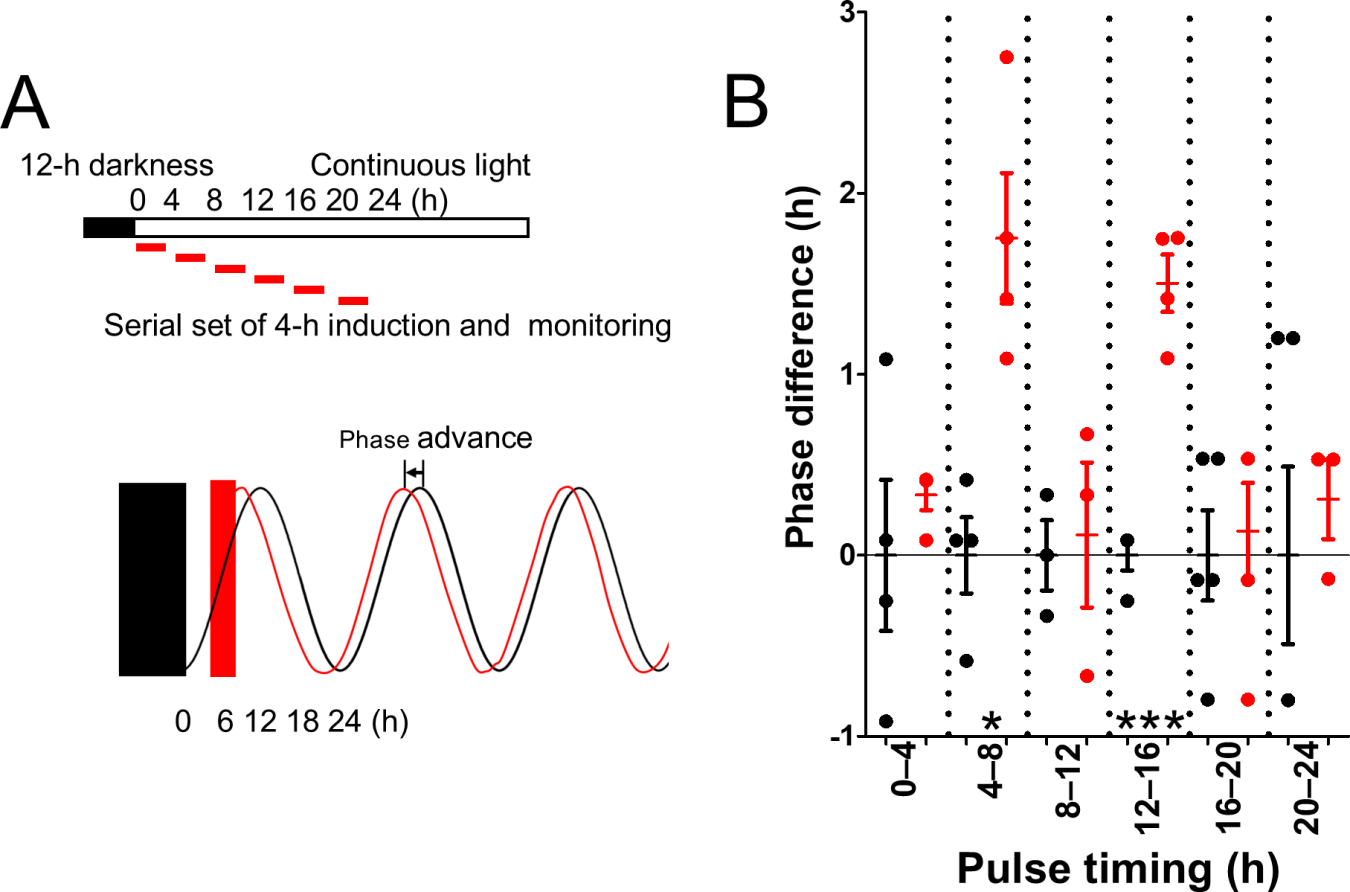
Circadian-phase advance by pulse induction of *ydeH*. (**A**) Experimental procedure and light conditions. Cultures grown under light were subjected to 12-h darkness (filled bar and rectangle). Red boxes indicate 4-h induction periods of *ydeH*, followed by IPTG removal via medium exchange. Bioluminescence monitoring was performed under continuous light. Phase-advanced rhythms resulting from induction between 4 and 8 h are shown in black (control strain) and red (*ydeH* strain). (**B**) Distribution of circadian-phase differences between *ydeH* and control strains. The vertical axis represents the circadian phase. Control averages (black circles) were set to 0 h; peak timings in *ydeH* strains are shown as red circles. Positive values indicate phase advances; negative values indicate delays. Error bars represent SEM. *, *P* < 0.05; ***, *P* < 0.001 (Student’s *t*-test, *n* = 3–5).

To examine which pulse timing of the circadian clock contributed to the phase delay during continuous light conditions, *ydeH* expression was induced by 4-h pulse treatments over the course of 0–24 h (Fig. 4A), following a protocol similar to that used for temporal induction of *yhjH* (Fig. 2). However, phase-advanced rhythms of about 1.5 h were observed by pulse timing for 0–4 h and 12–16 h (Fig. 4B), rather than the phase delay investigated in the continuous induction of *ydeH* (Fig. 3C). Although this phase-advance effect was small, it may have resulted from a shift in c-di-GMP levels from higher to lower ranges (Fig. S3).

### Exogenous c-di-GMP delays resetting of the circadian clock

In bacterial species (*Vibrio splendidus*, *Streptococcus mutans*, and *Staphylococcus aureus*), exogenously administered c-di-GMP inhibits biofilm formation (34–36). However, whether such administered c-di-GMP influences behaviors in cyanobacteria remains unknown. Accordingly, we examined the effect of c-di-GMP on the cyanobacterial circadian clock. In the *S. elongatus* bioluminescence reporter strain, we administered c-di-GMP and c-di-AMP (the cyclic dimer of AMP) at final concentrations of 8 µM each, corresponding to the dose range previously used in those bacterial species, while the intracellular c-di-GMP concentration in *S. elongatus* before the administrations was 120.3 ± 16.6 µM (mean ± SD, *n* = 3).

**Fig 5:**
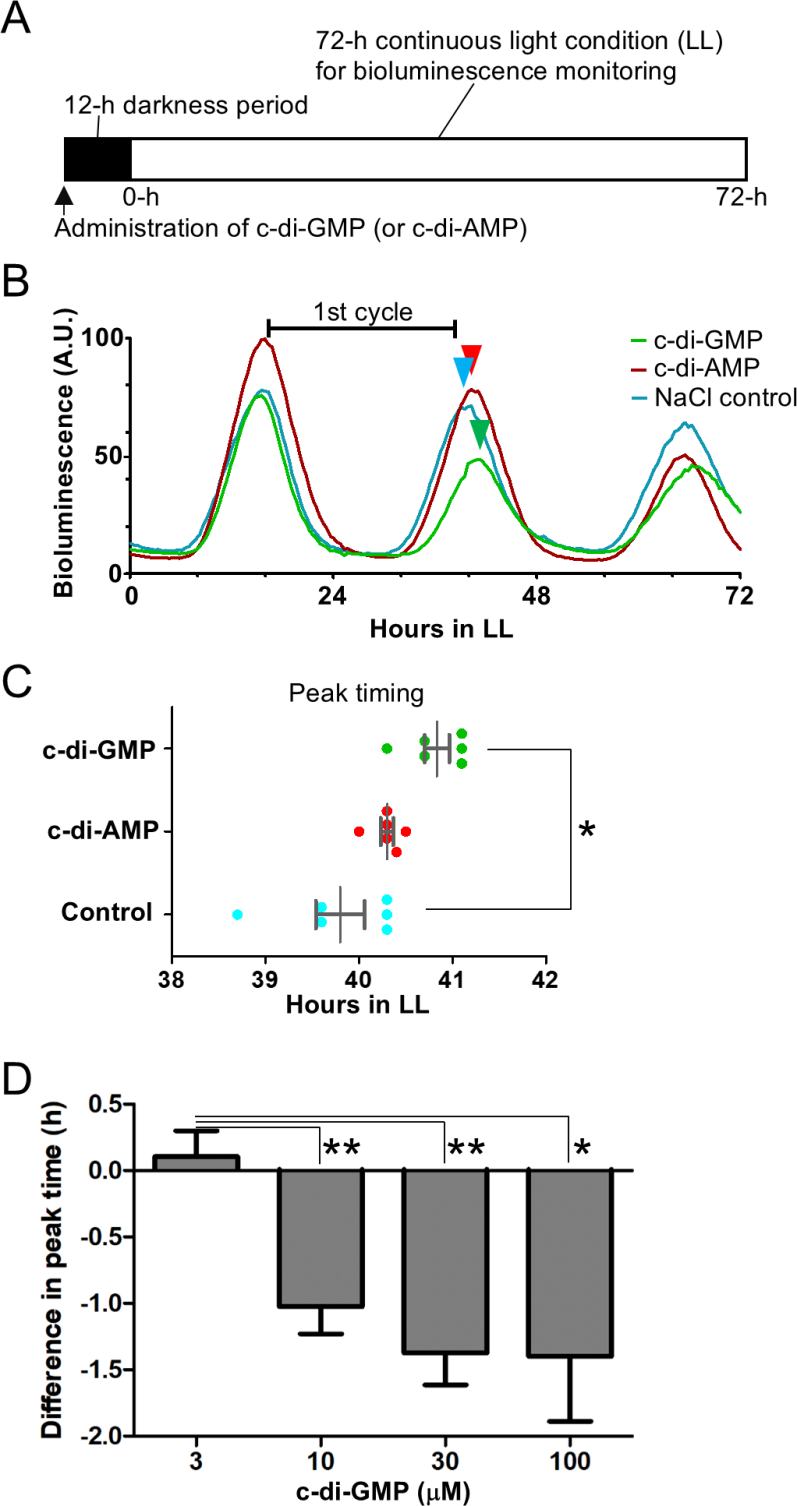
Effects of c-di-GMP administration during the dark period on circadian phasing. (**A**) Experimental procedure and light conditions. NaCl solutions containing 8 µM c-di-GMP or c-di-AMP were administered to liquid cultures at the onset of the dark period. Bioluminescence monitoring was performed under continuous light for the subsequent 72 h (LL). (**B**) Bioluminescence rhythms of *S. elongatus* cultures treated with exogenous cyclic dinucleotides. Bars indicate the duration of the first circadian cycle, which was used for quantitative comparison. Arrowheads mark peak timings of bioluminescence rhythms, reflecting the effects of c-di-GMP, c-di-AMP, and mock control (NaCl). (**C**) Distribution of second peak phases in bioluminescence rhythms (*n* = 6). (**D**) Dose-dependent effects of c-di-GMP on the circadian phase. Final concentrations were 0, 3, 10, 30, and 100 µM (*n* = 4). Error bars represent SEM. *, *P* < 0.05; **, *P* < 0.01 (Student’s *t*-test).

To assess the effects of cyclic di-nucleotide administration, cultures were treated with c-di-GMP, c-di-AMP, or a mock solution (NaCl) just before the resetting of the circadian clock under a 12-h dark condition, which was followed by bioluminescence monitoring under continuous light (Fig. 5A).

In the c-di-GMP-administered cultures, the second and third bioluminescence peaks were delayed by approximately 1 h compared to the controls, as shown in Fig. 5B and C.

In cultures administered with c-di-GMP, the circadian periods based on the first cycle were significantly different from those of the controls (*n* = 8; *P* < 0.05; Fig. S4). However, the periods calculated from the intervals between the second and third peaks (second cycle) and between the third and fourth peaks (third cycle) were approximately 25 h and showed no significant differences between the c-di-GMP-treated and control cultures. Specifically, the periods between the second and third peaks were 25.00 ± 0.31 h for the controls and 24.70 ± 0.20 h for the c-di-GMP-treated cultures (*n* = 8), and those between the third and fourth peaks were 24.63 ± 0.35 h and 24.68 ± 0.33 h, respectively (*n* = 8).

Overall, these results indicated that c-di-GMP administration during 12 h of darkness fine-tuned the phase of the circadian rhythm but did not affect the period after the second circadian peak timing (Fig. 5C). Although the addition of c-di-AMP elicited a smaller phase delay, no significant difference was detected (Fig. 5B and C).

Furthermore, when we assessed the effects of c-di-GMP administration under light conditions, neither the phase nor the period was altered (Table 3), suggesting that the response depends on the dark period or on transitions in light conditions.

**TABLE 3:** Effects of light conditions and nucleotides on circadian-phase differences^*a*^

Nucleotide	Condition	Concentration in the culture (µM)	Phase difference (h)	SEM (h)	*P* value
c-di-GMP	Dark	60	−0.9	0.2	0.0288*
c-di-GMP	Light	8	0.0	0.3	0.4982
c-di-AMP	Light	8	−0.1	0.1	0.3364
GMP	Dark	30	−0.3	0.3	0.3041
GTP	Dark	30	0.2	0.3	0.3539

^*a*^ Asterisk shows statistical significance. Student’s *t*-test (paired) (*n* = 5).

### Threshold concentration of c-di-GMP required for circadian-phase delay

Next, we investigated the relationship between the concentration of c-di-GMP and the circadian-phase delay by exposing *S. elongatus* reporter cells to c-di-GMP at concentrations ranging from 3 to 100 µM and comparing the phases of the bioluminescence rhythms. When 3 µM c-di-GMP was added, the phase was nearly identical to that of the control (Fig. 5D). However, concentrations between 10 and 100 µM induced a statistically significant phase delay of approximately 1 h, similar to the previously described 8 µM treatment (Fig. 5C and D).

To determine whether the observed dose-response exhibited linearity or a threshold effect, the correlation between c-di-GMP concentration and phase difference was statistically analyzed within the range of 3–30 µM c-di-GMP. However, no significant correlation was found (Pearson’s *r*^2^ = 0.71). Circadian-phase delay was observed above 3 µM and appeared to plateau between 30 and 100 µM. In summary, these results indicated that a c-di-GMP concentration threshold was reached in this concentration range rather than a concentration dependency.

## DISCUSSION

### Induction of the *yhjH* gene extends circadian periods

In cyanobacteria, the bacterial signaling molecule c-di-GMP is involved in biofilm formation, motility, heterocyst development, cell size control, phototaxis, and flocculation (37). In this study, to investigate the function of c-di-GMP in the circadian clock of cyanobacteria, we engineered *S. elongatus* strains harboring *yhjH* and *ydeH* genes. First, we found that the circadian rhythm of the *yhjH* strain (PDE-expressing strain) showed a 0.6-h extended circadian period (Fig. 1). Moreover, pulse induction of *yhjH* during the subjective evening or late night delayed the circadian phase by 1 h (Fig. 2), suggesting a time-dependent sensitivity of the clock to c-di-GMP degradation. Since KaiC binds GTP *in vitro* (38), we tested whether the dimer c-di-GMP directly modulates KaiC activity using reconstitution assays (Fig. S5). However, dissolved c-di-GMP did not affect the KaiB-KaiC binding rhythm (Fig. S5A [24]), nor did it affect KaiC phosphorylation rhythms in a consistent manner (Fig. S5B [39]). These findings suggest that c-di-GMP influences the circadian clock through mechanisms independent of direct binding to Kai proteins.

GTP, the substrate for c-di-GMP synthesis, may influence the circadian period and phase, as KaiC binds GTP *in vitro* (38). Although the link between GTP and the circadian clock remains unclear, measuring cellular GTP levels in the *yhjH*-induced strain may help clarify interactions among GTP, c-di-GMP, and clock regulation. Because c-di-GMP synthesis consumes GTP and the degradation may replenish it, fluctuations in GTP levels may affect the circadian clock. GTP is also required for ribosome and rRNA synthesis (40), and such changes may impact the circadian period (41). Taken together, our findings suggest that c-di-GMP modulates the circadian rhythm indirectly.

KaiC constitutes a transcription/translation feedback loop together with circadian transcription factors, such as Pex (21, 30, 42, 43). Therefore, c-di-GMP may act on transcription factors *in vivo*. In *Anabaena* sp. strain PCC 7120, the transcriptional regulatory system includes the c-di-GMP binding effector CdgR, which regulates cell size control (16). The *S. elongatus* genome also harbors a homolog of *cdgR*; however, its function remains unknown (16). This putative CdgR may sense c-di-GMP levels and control *kaiABC* transcription. In addition, translation and proteolysis contribute to the maintenance of circadian periods (41, 44), suggesting that c-di-GMP may modulate these activities directly or indirectly.

Under long-term or pulse induction conditions, cellular c-di-GMP levels were comparable between the *yhjH* strain and the control (Fig. S2B and S3A). However, the *yhjH* strain exhibited a wider range of c-di-GMP levels, emphasizing the experimental challenges in the quantification of c-di-GMP at lower concentrations. This may also suggest strong internal control of c-di-GMP levels at lower concentrations. More frequent sampling time points may help detect transient decreases in c-di-GMP.

### Elevated c-di-GMP levels in the *ydeH* strain delay circadian-phase resetting

Induction of *ydeH* expression at the onset of light conditions increased c-di-GMP levels and caused a phase delay of approximately 6 h (Fig. 3; Fig. S2). This delayed response was similar to the phenotype exhibited by a strain deficient in the gene for the CmpR transcription factor (45). CmpR is a transcriptional activator for the bicarbonate transporter operon and is required for stress adaptation to low CO_2_ and high light environments (46). The colony size and color of the *ydeH* strain were smaller and pale green, respectively, which was similar to a *cmpR* mutant (Fig. S6; 45), however, it is unknown whether CmpR is a component associated with the phase delay found in the *ydeH* strain.

c-di-GMP increases at the onset of the dark period for 20 min but decreases upon light exposure ([33] Fig. S2A). The phase delay (1.5 h) observed after the addition of c-di-GMP during the dark period but not at the onset of the light conditions (Table 3) may suggest that the cells are more active in the degradation of c-di-GMP at the onset of the light conditions; however, such degradation activity was not examined here. The *S. elongatus* phosphodiesterase, namely, SL2 (*Synechococcus* LOV protein 2), degrades c-di-GMP *in vitro* under blue light conditions (19). The biological function of this protein is not yet known, but it is intriguing in the context of the regulation of the circadian period length and phase.

Most of the genes in *S. elongatus* are controlled by the circadian clock and diel light (47, 48). Twenty genes in the strain are associated with c-di-GMP metabolism, and we summarized these gene-expression timings (Table S1). Results showed that these expression peak timings covered most of circadian time (CT) except CT 4–CT 9 (subjective late morning and afternoon, Table S1), suggesting the physiological importance of circadian control of these gene expressions. Among these genes, it was found that six genes were expressed during the dark period (Table S1). These six genes may relate to the decrease in c-di-GMP during the dark–light transitions. Future study is required to establish the role of these c-di-GMP-associated genes in the circadian phasing.

### Administration of c-di-GMP in the dark period delays the circadian phase

c-di-GMP administration experiments have been conducted in *Vibrio splendidus*, *Streptococcus mutans*, and *Staphylococcus aureus* (34–36); however, the mechanisms by which the administered c-di-GMP acted extracellularly via either extracellular receptors or through internal uptake are not well understood. Herein, when *S. elongatus* was cultured in media with c-di-GMP at 8 µM, the circadian-phase delay occurred (Fig. 5). In the above virulent bacterial strains, biofilm formation was inhibited when cultured in media containing 10–50 µM c-di-GMP. Thus, the sensitivity to exogenous c-di-GMP in *S. elongatus* was in a range similar to these previous reports.

The average intracellular c-di-GMP concentration in *S. elongatus* was 120.3 µM (SD = 16.6, *n* = 3), far exceeding the 8 µM threshold previously shown to modulate the circadian phase upon exogenous application (Fig. 5). *S. elongatus* cells may uptake c-di-GMP from the medium, or the action of c-di-GMP in the medium may be transduced into the cell by an unknown receptor, such as receptors involved in chemotaxis (49). Further research is needed to understand the mechanism by which administered c-di-GMP regulates the circadian rhythm phase in *S. elongatus*.

### Conclusions

In this study, physiological and molecular genetic analyses demonstrated that intracellular c-di-GMP levels influenced the period, phase, and amplitude of circadian rhythms in *S. elongatus* PCC 7942. The endogenous genes controlling c-di-GMP metabolism and the precise mechanisms by which these occur remain unresolved. Therefore, future molecular genetic investigations are crucial for elucidating the pathways that govern c-di-GMP signaling and for defining its role in the circadian systems of cyanobacteria. The mechanism(s) by which c-di-GMP levels influence the circadian clock in *S. elongatus* shall be the subject of future investigations.

## MATERIALS AND METHODS

### Chemicals

C-di-AMP was synthesized chemically (purity >91% [50]) and c-di-GMP was purchased from BioLog (Bremen, Germany; purity >95%). Other reagents were purchased from Fuji Film Wako Pure Chemical (Osaka, Japan) and used for cell culture and bioluminescence monitoring. Deionized water was filtered by membrane filtration (hydrophilic nylon membrane of 0.22 μm pore size; Starlab, Barcelona, Spain) and sterilized by autoclaving. Ethanol, phenol, chloroform, and isoamyl alcohol were used in extractions, and methanol was used during liquid chromatography tandem electrospray ionization mass spectrometry (LC/ESI-MS/MS); all reagents were purchased from Fuji Film Wako. Xanthosine 3′,5′-cyclic monophosphate (cXMP) was procured from Sigma-Aldrich (purity >95%; St. Louis, USA) and was used as an internal standard during quantification of cellular c-di-GMP (51).

### Bacterial culture and media

*E. coli* competent DH5α cells were purchased from Takara Bio (Kusatsu, Shiga, Japan). NUC42 strain, a cyanobacterial derivative of *S. elongatus* PCC 7942, was cultivated at 30°C in modified BG-11 liquid medium.

### Bioluminescence rhythm measurement

Bioluminescence monitoring in *S. elongatus* was carried out as described in a previous report (32). *S. elongatus* NUC42 was cultivated with BG-11 liquid medium to a cell density of 0.4 (optical density at 730 nm [OD_730_]). The culture was entrained to three cycles of 12-h light:12-h dark. The light intensity was 20.7 μmol/m^2^/s. After entrainment, the culture was diluted with BG-11 liquid medium to an OD_730_ of 0.1. The nucleotides and inducer molecule isopropyl-β-D-thiogalactopyranoside (IPTG) were added to the culture. If necessary, the cultures were centrifuged at 16,000 × *g* for 2 min to remove the media containing IPTG. The precipitated cells were resuspended in fresh media.

Such cultures were placed in 0.5 mL volume plastic containers. As a luminescent substrate, 20 µL of 1% *n*-decanal was placed in a 50 μL volume plastic container. These were placed in a small glass petri dish, and it was covered with a piece of paraffin film (Parafilm; Bemis Flexible Packaging, Chicago, IL, USA). The luminescence rhythm was measured at 30°C under continuous light, and the light intensity was 24.4 μmol/m^2^/s.

### Establishment of *S. elongatus* strains that express *E. coli* genes, *yhjH*, or *ydeH*

To construct the plasmid, pTS2Ktrcp-*yhjH*, the following linker-primers were used: 5′-GGTACCCGGGGATCCATAAGGCAGGTGATACAGCGA-3′ and 5′-CGACTCTAGAGGATCTTATAGCGCCAGAACCGCCGT-3′, and to construct the plasmid, pTS2K*trc*p-*ydeH* plasmid, 5′-GGTACCCGGGGATCCATCAAGAAGACACGGAAATT-3′ and 5′-CGACTCTAGAGGATC- TTAAACTCGGTTAATCATT-3′ were used. The second and stop codons are underlined, respectively. The open-reading frame of the genes was amplified from *E. coli* genomic DNA by PCR with the linker-primers. The amplified DNAs were inserted into the *Bam*HI site of the plasmid vector pTS2K*trc*p (52). The restriction enzyme *Bam*HI and In-Fusion HD Cloning Kit (Takara Bio) were used according to the manufacturer’s instructions. The plasmids obtained were used to transform strain NUC42, a representative cyanobacterial bioluminescent transformant strain (32). The resulting expression-inducible strains for *yhjH* and *ydeH* were designated YCC102 or YCC108, respectively. The plasmid vector pTS2K*trc*p was also used to transform NUC42, and the obtained transformant was designated YCC100 and used as the control strain.

QuikChange Site-Directed Mutagenesis Kit (Stratagene) was used for *in vitro* mutagenesis of pTS2K*trc*p-*yhjH* and pTS2K*trc*p-*ydeH* with oligonucleotide primers as follows: for *yhjH*^ALA^, 5′-GGTTAATGGCCGTG(GCGCTAGCA)ACGGTGGTCACGCA-3′, and for *ydeH*^GGAAF^, 5′-CGGTTTATCGCTAC(GGGGGCGCAGCATTT)ATCATTATTGTC-3′. Both the above and complementary oligonucleotides were used according to the manufacturer’s instructions. Underlines show nucleotides targeted for the mutagenesis. The triplets of nucleotides corresponding to amino acids are in parentheses. The triplets on *yhjH* are the 48th to 50th residues, Ala-Leu-Ala. The triplets in *ydeH* mutations correspond to the 206th to 210th amino acid residues, Gly-Gly-Ala-Ala-Phe.

### Quantitative analyses of c-di-GMP

Extraction and quantitative analyses by LC/ESI-MS/MS were conducted according to Kameda et al. (33). *Synechococcus* cultures, with an OD_730_ between 0.2 and 1.0, were entrained under three light–dark cycles (light:dark = 12:12 h). At the end of the third dark period, which was set to 0 h, IPTG was added to the cultures. Cells were collected at 0, 2, and 4 h, and the number of cells was normalized to 3.9 × 10^7^ cells. Afterward, cXMP was added to the cell suspension as an internal calibration standard. c-di-GMP was subsequently extracted via boiling and purified using water-saturated acidified phenol (33).

### Circadian period analysis and statistical analysis

Circadian bioluminescence rhythms were analyzed to determine circadian periods by using BioDare2 (https://biodare2.ed.ac.uk; 53, 54). Statistical analyses were performed using Prism (GraphPad, San Diego, USA).

## SUPPLEMENTAL MATERIAL

Supplemental figures and tables jb.00574-25-s0001.pdf Figures S1 to S6 and Table S1.

ASM does not own the copyrights to Supplemental Material that may be linked to, or accessed through, an article. The authors have granted ASM a non-exclusive, world-wide license to publish the Supplemental Material files. Please contact the corresponding author directly for reuse.
