# Supplementary material for: Effects of the signaling molecule cyclic-di-GMP on cyanobacterial circadian rhythm in Synechococcus elongatus PCC 7942
Source: J Bacteriol. 2026 Feb 27;208(3):e00574-25. doi: 10.1128/jb.00574-25 (PMC13001216; doi:10.1128/jb.00574-25)
Supplement: Supplemental figures and tables — Figures S1 to S6 and Table S1. [file jb.00574-25-s0001.pdf]

Supplementary material for:

Effects of the signaling molecule cyclic-di-GMP on cyanobacterial circadian rhythm in *Synechococcus elongatus* PCC 7942

Contents: Figures S1 to S6; Table S1

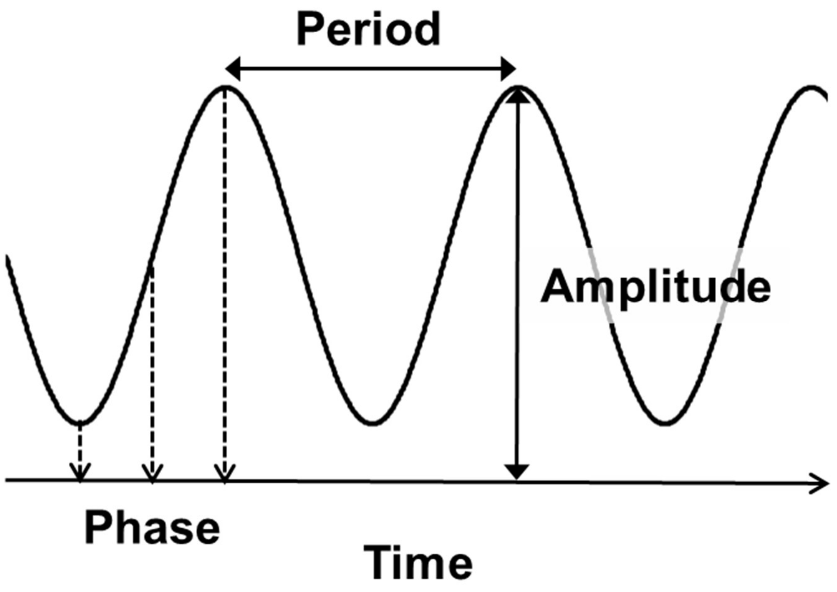

Fig. S1. Three parameters investigated in regard to circadian bioluminescence rhythm.

Period, the time of each cycle of the bioluminescence circadian rhythm. For example, the double-sided arrow represents the circadian period based on the time between two peaks. Phase, each representative timing of the rhythm. Horizontal dashed arrows represent phases of trough, half level, and peak. In this study, the timings of peaks were compared to a representative phase. Amplitude, bioluminescence level at the peak is represented by a two-sided vertical arrow.

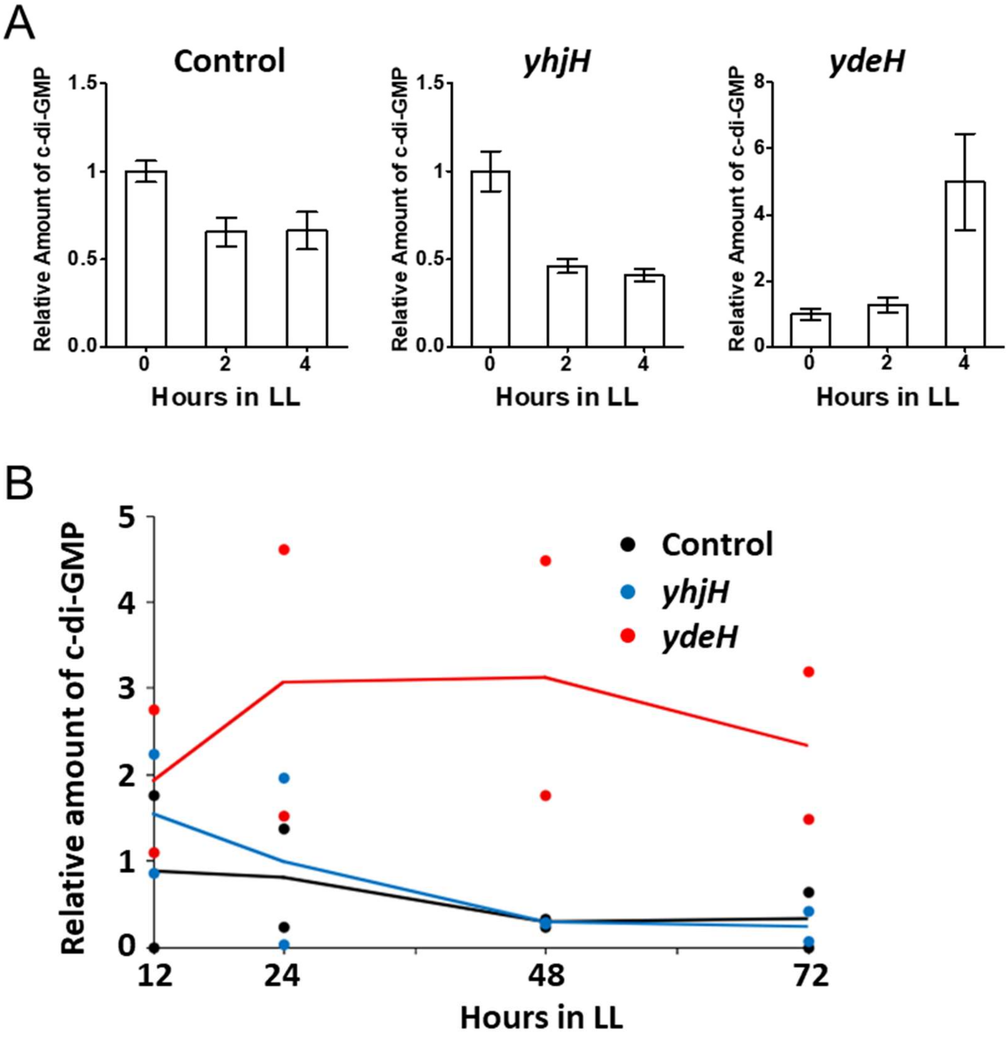

Fig. S2. Changes in intracellular c-di-GMP levels in *S. elongatus* strains following gene induction.

(A) Relative amounts of c-di-GMP measured under continuous light conditions (0 to 4 h). Control *S. elongatus* strain (*trc* promoter without either the *yhjH* gene or *ydeH* gene); *S. elongatus* strains harboring P*trc*::*yhjH* or P*trc*::*ydeH.* Experimental procedures and light conditions were identical to those in Figs. 1B and 3B. Hour 0 was defined as the onset of light immediately before induction. Intracellular c-di-GMP levels were normalized to those at hour 0. Data are presented as means ± SEM (n = 3 or 4).

(B) Relative amounts of c-di-GMP measured under continuous light conditions over 72 hours. Circles and lines represent individual measurements and their mean values, respectively (n = 2).

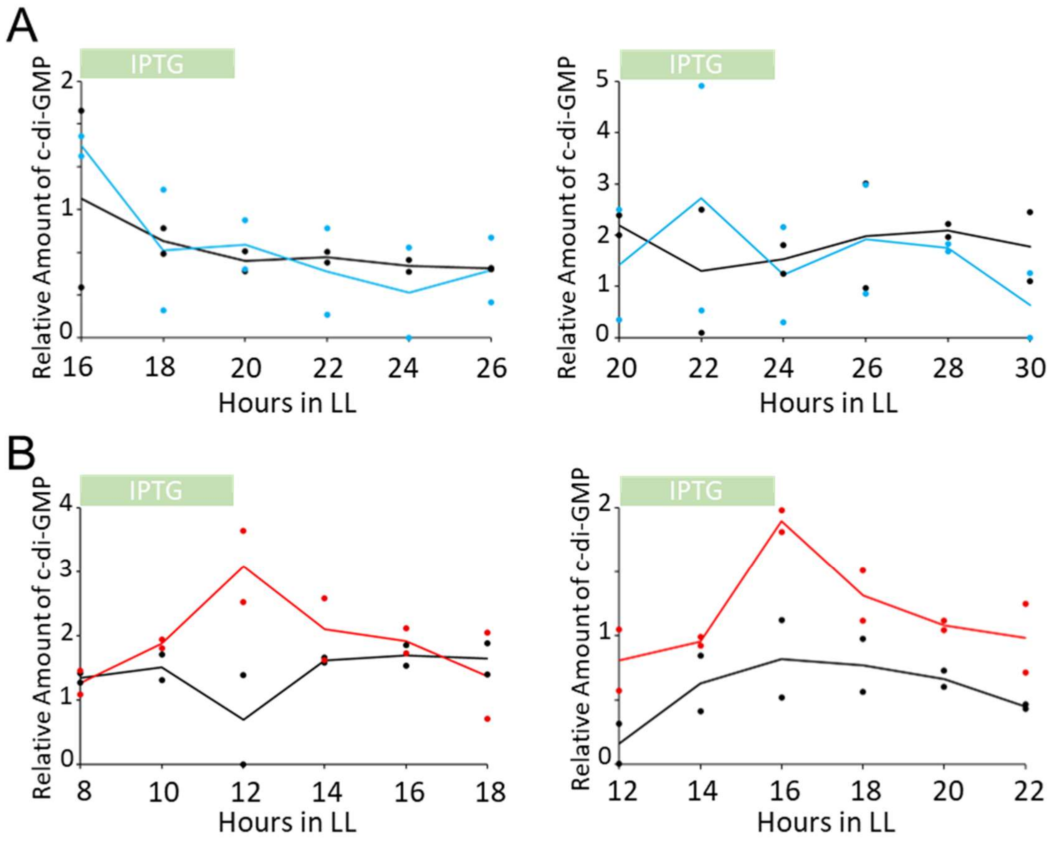

Fig. S3. Cellular c-di-GMP levels in response to IPTG administration for gene induction.

(A) c-di-GMP levels during induction of P*trc::yhjH* (blue). Left: IPTG was added to cyanobacterial cultures for 4 h (16–20 h in LL) and then removed. Right: c-di-GMP levels during and after 4 h of IPTG administration (20–24 h). These experiments correspond to the pulse-induction conditions shown in Fig. 2B. Variations in c-di-GMP levels between control and *yhjH* strains remained within the same range.

(B) c-di-GMP levels in P*trc::ydeH* (red). Left: IPTG induction from hour 8 to 12. Right: IPTG induction from hour 12 to 16. c-di-GMP levels increased during IPTG administration, reaching more than 2-fold higher than control levels, and declined after IPTG removal. This transient elevation may have contributed to the phase advance observed in Fig. 4. Experimental procedures and light conditions are as described in Fig. 2B. Hour 0 corresponds to the onset of light. Pale green boxes above each panel indicate IPTG administration timing. Circles and lines represent individual c-di-GMP measurements and their mean values, respectively (n = 2).

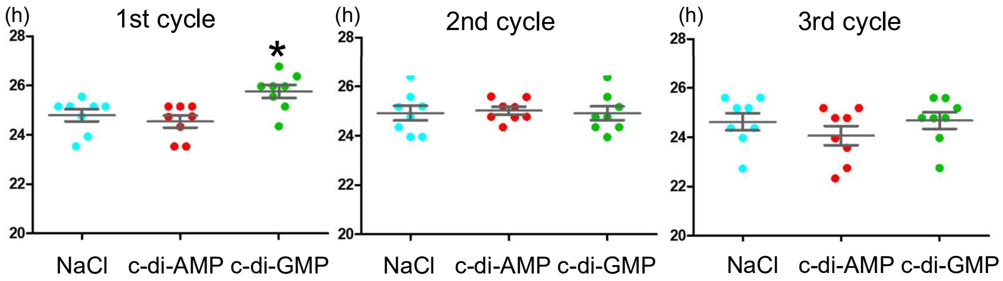

Fig. S4. Distribution of peak timing across the first, second, and third circadian cycles.

Cyclic dinucleotides were administered to *S. elongatus* liquid cultures at a final concentration of 8 μM. Data are presented as mean ± SEM (n = 8). Statistical significance was determined by one-way ANOVA; $P < 0.05$.

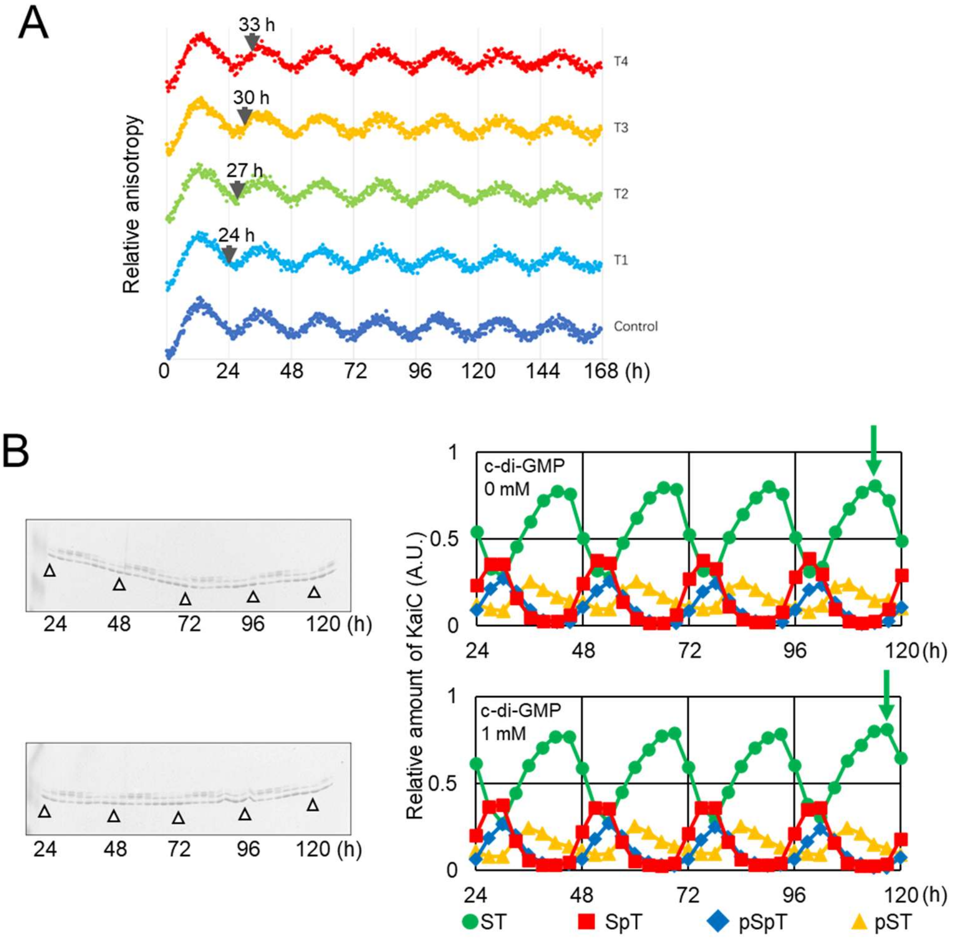

Fig. S5. *In vitro* reconstitution assays for dynamic interactions among Kai proteins.

(A) *In vitro* oscillation monitored by anisotropy assay using fluorophore-labeled KaiB and DNA. The vertical axis indicates relative anisotropy values reflecting KaiB–KaiC binding. c-di-GMP (250 μM) was administered at various time points, as indicated by arrowheads. (B) The KaiC phosphorylation cycle *in vitro*. Upper and lower panels show KaiC phosphorylation states under 0 mM and 1 mM c-di-GMP, respectively. (Left) Representative gel images of KaiC bands corresponding to phosphorylation states; open triangles mark lanes sampled every 24 h. (Right) Circadian profiles of KaiC phosphorylation based on band intensity; arrows indicate the fourth peak in the ST band, reflecting a difference in peak timing due to the influence of dissolved c-di-GMP on the period length in the KaiC phosphorylation cycle *in vitro*. Reaction mixtures contained KaiA (0.04 mg/mL), KaiB (0.04 mg/mL), and KaiC (0.2 mg/mL) and were incubated at 30 °C. Samples were collected every 3 h using an autosampler and analyzed by SDS–PAGE. Phosphorylation states: ST, fully dephosphorylated KaiC; SpT, phosphorylated Thr432; pST, phosphorylated Ser431; pSpT, dual phosphorylation at Ser431 and Thr432. Incubation buffer: 20 mM Tris-HCl (pH 8.0), 0.15 M NaCl, 0.5 mM EDTA, 5 mM $MgCl_2$, 1 mM DTT, and 1 mM ATP. Period lengths were 23.7 h (0 mM c-di-GMP) and 24.0 h (1 mM c-di-GMP), indicating a 0.3 h extension of the circadian period by c-di-GMP. Fitting error was ±0.02 h.

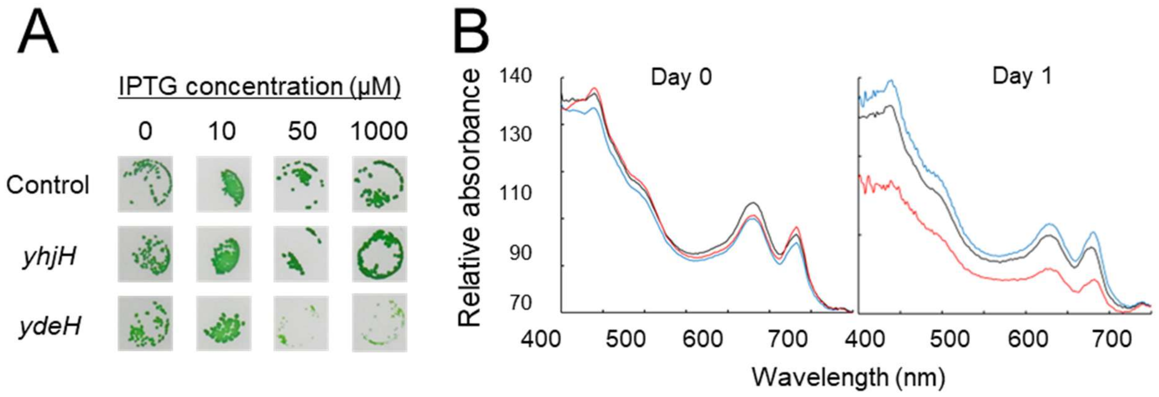

Fig. S6. Analysis of *S. elongatus* cells with altered c-di-GMP levels.

(A) Colony formation on solid medium containing the inducer IPTG. Expression of *yhjH* and *ydeH* was used to decrease and increase intracellular c-di-GMP levels, respectively. Ten microliters of medium containing approximately 50 cells were spotted onto solid plates supplemented with IPTG at concentrations ranging from 0 to 1000 μM.

(B) Whole-cell absorption spectra of *yhjH*- and *ydeH*-induced cells cultured in liquid medium. IPTG was added on day 0, and spectral measurements were taken on day 1. Cell density was estimated by absorbance at 730 nm and used to normalize the spectra.

Table S1 Expression levels and circadian/diel timing of c-di-GMP-related genes in *Synechococcus elongatus* PCC 7942.

Gene ID	Expression (log 2)	CT	Dark/Light	c-di-GMP related domain	Other domains	Annotation
Synpcc7942_0490	0.12	0	0.3	GGDEF	PAS/PAC	Hypothetical protein
Synpcc7942_2192	0.45	0	0.8	GGDEF	HAMP	Hypothetical protein
Synpcc7942_1382	0.18	2	0.1	GGDEF/EAL	PAS/PAC, MASE	Hypothetical protein
Synpcc7942_2193	0.06	2	0.5	HD-GYP	GAF	Hypothetical protein
Synpcc7942_0151	0.10	3	0.5	GGDEF	CheY-like, asp_protease, Wing_hlx_DNA_bd	Two-component response regulator
Synpcc7942_1298	0.12	10	2.2	GGDEF	GAF, CHASE	Hypothetical protein
Synpcc7942_0816	0.13	11	0.4	GGDEF	PAS	Hypothetical protein
Synpcc7942_1811	0.28	12	1.2	GGDEF	PAS/PAC	Hypothetical protein
Synpcc7942_2534	0.09	13	0.5	GGDEF/EAL	GAF, PCAD	Hypothetical protein
Synpcc7942_1355	0.33	14	0.3	GGDEF/EAL	PAS/PAC, CheY-like, PYP	SL2, Cao et al., 2010. two-component response regulator.*
Synpcc7942_0122	0.10	15	0.5	EAL	Not determined	Hypothetical protein
Synpcc7942_0188	0.12	16	0.9	GGDEF/EAL	PAS/PAC, LOV	SL1, Cao et al., 2010.*
Synpcc7942_1158	0.28	17	0.6	GGDEF/EAL	PAS/PAC, GAF	Hypothetical protein
Synpcc7942_2519	0.20	19	0.7	GGDEF/EAL	PAS, MASE	Hypothetical protein
Synpcc7942_2096	0.21	20	4.6	GGDEF	GAF, PCAD, CBS	Hypothetical protein
Synpcc7942_1148	0.36	21	3.5	HD-GYP	GAF	Hypothetical protein
Synpcc7942_2535	0.33	22	3.2	GGDEF	PAS/PAC, GAF	Hypothetical protein
Synpcc7942_1643	0.12	22	0.7	GGDEF	GAF	Hypothetical protein
Synpcc7942_1716	0.21	22	1.6	GGDEF/EAL	PAS/PAC, LOV	Hypothetical protein
Synpcc7942_1859	0.17	23	0.5	GGDEF/EAL	PAS/PAC, LOV	Hypothetical protein

Expression values represent relative transcript abundance under continuous light conditions. CT (circadian time) indicates the estimated endogenous phase under constant light, where CT 0 and CT 12 correspond to subjective dawn and dusk, respectively. Dark/Light values represent the ratio of gene expression level of diel dark and light conditions. Ratio > 1: The gene's expression level is higher in the dark condition. Data were compiled from previously published studies: Ito et al. (2009), *Proc. Natl. Acad. Sci. USA* **106**, 14168–14173; Vijayan et al. (2009), *Proc. Natl. Acad. Sci. USA* **106**, 22564–22568. Genes marked with an asterisk (*) were additionally reported in Cao et al. (2010), *Photochem. Photobiol.* **86**, 606–611.
